# Pathways to mental health care for patients with severe mental illness in Tunisia

**DOI:** 10.11604/pamj.2019.34.118.19661

**Published:** 2019-10-29

**Authors:** Houyem Khiari, Uta Ouali, Yosra Zgueb, Ali Mrabet, Fethi Nacef

**Affiliations:** 1 Faculty of Medicine of Tunis, University of Tunis El Manar, Tunis, Tunisia; 2 Preventive Medicine Department, Tunis, Tunisia; 3 Department Psychiatry A, Razi Hospital, Tunis, Tunisia; 4 Health Military Directorate, Bab Saadoun, Tunis, Tunisia

**Keywords:** Severe mental illnesses, pathway, mental health, consultation delay, Tunisia

## Abstract

**Introduction:**

Schizophrenia, bipolar disorder and schizoaffective disorders are severe mental illnesses (SMI) associated with high levels of co-morbid psychopathology and premature mortality. Reducing delays in accessing services and providing early intervention are key strategies in preventing morbidity and mortality associated with these diseases. The pathways to psychiatric care have been studied in many countries worldwide. To the best of our knowledge, no study on this subject has so far been conducted in Tunisia. The purpose of the present study was to understand the pathways of care adopted by patients, to determine the care delay and to explore the relationship between delayed consultation and socio-demographic and clinical variables.

**Methods:**

This is a cross-sectional descriptive study conducted at the Department Psychiatry A of Razi Hospital including patients with SMI consulting the outpatient clinic between January and March 2018. Data was collected by one medical investigator who conducted face-to-face interviews with patients using a questionnaire based on the World Health Organization's "Pathway Questionnaire". Data analysis was done using the SPSS software version 17. A multivariate analysis was performed to study the relation between delayed consultation and socio-demographic and clinical variables.

**Results:**

A total of 232 patients responded to the questionnaire. The average age was 41.3 years ± 10.1 and the gender ratio was 1.2. More than the third of the study population consulted a traditional healer in the first place and sixty percent of the patients had recourse to a medical doctor. The average consultation delay was 15 months (±23.0) with a median of 6 months. The delay was more than 6 months in around half of the cases. The symptoms that motivated the first consultation were hallucinations, sleep disorders and aggressive behavior. The main reason of delayed consultation was lack of knowledge about psychiatric symptoms followed by illness beliefs and insidious onset of the illness. The multivariate analysis showed a significant relationship between aggressive behavior and non-delayed consultation.

**Conclusion:**

The principal recommendations are to strengthen public education and awareness about SMI in the Tunisian population and to implement an early detection program of these disorders.

## Introduction

Mental disorders are an important public health issue and one of the leading causes of disability [1,2]. The WHO "Global burden of disease" study has classified psychoses as one of the top ten causes of handicap in the world [1,3,4]. Schizophrenia, bipolar disorder and schizoaffective disorders are severe mental illnesses (SMI) associated with high levels of co-morbid psychopathology and premature mortality [5,6]. In addition, SMI has a significant impact on individual, family, social and professional functioning [7]. In recent years, early intervention for these diseases has become a very important area of development in the mental health community [8]. In fact, research has suggested that delayed access to mental health services and treatment in schizophrenia and bipolar disorder is associated with slower or less complete recovery, and increased risk of relapse and poorer prognosis [9-11]. Reducing delays in accessing services and providing early intervention are key strategies in preventing morbidity associated with SMI [12,13]. The pathway to mental health care can help to understand the delay in attending consultation and treatment. In fact, the concept of "pathways to care" describes the sequence of contacts with individuals and organizations prompted by the distressed person's efforts and those of their relatives to seek help as well as the help that is supplied in response to such efforts [14]. The health care system in Tunisia is comprised of a public and a private sector. The public sector is divided into three levels of care: (i) a large network of primary health care centers which are distributed evenly throughout the country, which are considered the main entry point into the public health care system, (ii) district and regional hospitals, and (iii) university-related hospitals and specialized institutions. The private sector consists of both private practice as well as outpatient and inpatient clinics [15,16]. Specialist mental health care services are mainly provided by university hospitals situated in the capital (Razi Hospital, a psychiatric specialty hospital) and along the coastline which makes their access more difficult for people living in the interior of the country [15,17]. This is one of the reasons why public mental health services in Tunisia have been organized by catchment or service areas. Every academic service is responsible for the provision of mental health care to the population and the training in mental health of primary care providers, especially GPs, within its defined geographical area [15]. Still, until now, public second- and first-line institutions offer only limited to no mental health care at all. Government funding for public health care has been unable to keep up with the increased demand (dual impacts of rapid population growth and health affecting lifestyle changes). Therefore, large parts of the population have turned increasingly to the expensive private sector to meet their health care needs [16].

Pathways to mental health care studies describe the help seeking behavior of people with psychological symptoms and provide a quick and reliable overview into how health care systems work [18]. The patterns and correlates of these pathways may also inform interventions that could facilitate their contact with mental health professionals and reduce the consultation delay and the duration of untreated psychosis [19]. The pathways to psychiatric care have been studied in many countries across the world [18]. In Africa, many surveys have been conducted in Nigeria [20], Ethiopia [21], Ghana [22], Uganda [23], KwaZulu - Natal [24] and South Africa [25].To the best of our knowledge, no study on this subject has so far been conducted in Tunisia. Therefore, the present work was conducted to fill this gap by exploring the pathways to psychiatric care adopted by patients in Tunisia. The purpose of the present study was to describe the socio-demographic characteristics of patients with SMI attending Razi Hospital, to understand the pathways of care adopted by these patients, to determine the care delay and to explore the relationship between delayed consultation and socio-demographic and clinical variables.

## Methods

**Study design and place:** this is a cross-sectional descriptive study conducted at the Department of Psychiatry A of Razi Hospital including patients with SMI consulting the outpatient clinic between January and March 2018. This department covers a geographical area including the governorate of Beja, a predominantly rural or semi-rural area and some districts of the Greater Tunis region, which is an urban area. Data collection was spread over the three-month mentioned period, preceded by a one-month test phase to test the questionnaire and train the investigating clinician.

**Study participants:** this study concerned all patients consulting the outpatient clinic of Department Psychiatry A of Razi Hospital (every Thursday of each week) during the mentioned period and responding to the inclusion criteria. Patients included in the present study had a diagnosis of schizophrenia, bipolar disorder or schizoaffective disorder according to the DSM 5 criteria, were clinically stabilized with no inpatient admission during the three months preceding the study intake. Initial access to psychiatric care at the psychiatry A department had to be less than 10 years in order to keep memory bias to a minimum. Patients gave their oral informed consent prior to inclusion in the study. The study was approved by the Ethics Committee of Razi Hospital.

**Data collection:** data was collected by one medical investigator (KH) who conducted face-to-face interviews with patients consulting the outpatient clinic of Department Psychiatry A of Razi Hospital during the mentioned period. The investigator interviewed the patients and any family member if they presented with the patient, to have better and clearer answers. A hetero questionnaire was used including a section on patient identification (age, gender, educational level, marital status, environment and socio-economic level) and a second part based on the World Health Organization’s "Pathway questionnaire". Data collection was also based on the medical file for some questions, mainly for psychiatric diagnosis, and to complete and cross check data related to the pathway to psychiatric care.

**The "Pathway Questionnaire" and consultation delay:** the "Pathway to Mental Health Questionnaire" designed by the World Health Organization (WHO) is used to describe the pathway to care and to determine the care delay in psychiatry. Pathways to care were measured using the encounter form which documents the carer from whom help is initially sought (the first pathway contact), and then the next carer (second pathway contact), a third contact etc. until reaching a psychiatric specialist contact. This measure of pathways to care has previously been used in international studies of common mental disorders and psychotic disorders [26,27]. Pathways to health care studies using the pathway questionnaire are a simple, rapid and inexpensive method and can be done with minimal resources. The questionnaire helps in analyzing health services use, identification of the sources of delay in attending the right care and finding out adequate mental health programs and strategies that would lead to better outcome [27]. For the purpose of the present study, the World Health Organization’s Pathway questionnaire was translated from English into Arabic language by a bilingual translator, and then back-translated into English by another bilingual translator. Translation and back-translation were harmonized with the help of one linguist and members of the research team. The final Arabic version of the questionnaire was tested and adapted following a pre-test phase with a limited sample of patients. The consultation delay was defined as the period between the onset of first symptoms of the mental illness and consultation in psychiatry. Based on the findings of several studies in the literature, a period of more than 6 month was considered as delayed in the present study [18,28-30].

**Data analysis:** data analysis was done using the SPSS software version 17. The categorical variables were described through percentages and the quantitative variables through the calculation of means, median and standard deviation. The Chi^2^ test and the Student’s T test were used respectively for the comparison of percentages and means. A multivariate analysis was performed to study the relation between delayed consultation and socio-demographic and clinical variables. The limit of significance was set at 0.05.

## Results

**Socio-demographic and clinical characteristics of study participants:** a total of 232 patients responded to the questionnaire. The average age was 41.3 years ± 10.1 (39.8 among men and 43,1 among women). The gender ratio was 1.2 (54.3% males and 45.7% females). Table 1 describes the gender specified socio demographic and clinical variables.

**Table 1:** Socio-demographic and clinical characteristics of study participants by gender (repartition in percentage)

Characteristics		Male	Female	Total
Level of education	Illiterate	3.0	4.3	7.3
	Primary	20.3	24.6	44.8
	Secondary	25.0	11.6	36.6
	Higher	6.0	5.2	11.2
Marital status	Single	39.2	21.1	60.3
	Married	12.9	17.7	30.6
	Divorced	2.2	5.6	7.8
	Widower	0.0	1.3	1.3
Area	Urban	43.5	38.8	82.3
	Rural	10.8	6.9	17.7
Socioeconomic level	Low	39.7	37.1	76.7
	Medium	14.2	8.6	22.8
	High	0.4	0.0	0.4
Diagnosis	Schizophrenia	36.2	23.3	59.5
	Bipolar disorder	10.3	16.8	27.2
	Schizoaffective disorder	2.6	1.3	3.9
	Other	5.2	4.3	9.5

Other: unspecified schizophrenia spectrum disorder or unspecified bipolar disorder

**Figure 1:**
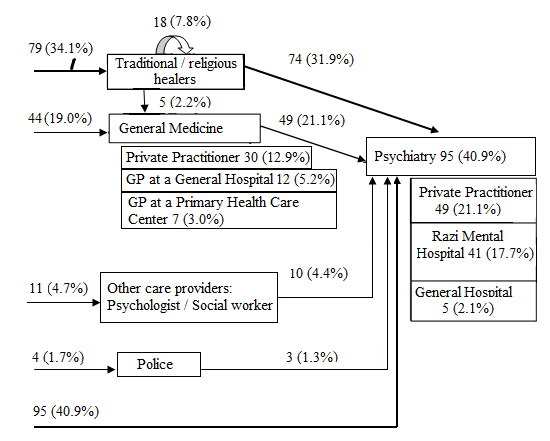
Pathways to mental health care in patients with SMI at Department Psychiatry A, Razi Hospital (repartition in number and percentage)

**Pathways to mental health care:** more than the third of the study population (34.1%) sought help at traditional healers or religious healers in the first place, and a fifth of the cases (19.0%) had recourse to general medicine (a private practitioner in 12.9%, a GP in a general hospital in 5.2% and a GP in a primary health care center in 3.0%). A psychiatrist was directly sought in 40.9% (a private practitioner in 21.1%, the Razi Hospital in 17.7% and a psychiatrist in a general hospital in 2.1%). Private sector was sought in a total of 34.0% of the cases (12.9% in general medicine and 21.1% to a psychiatrist in the private sector) (Figure 1).

**Figure 2:**
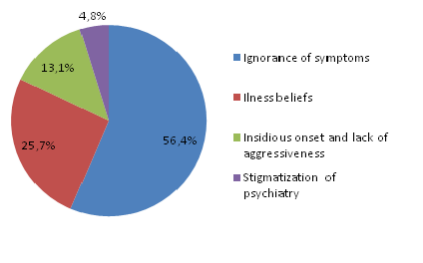
Reasons of delayed consultation in patients with SMI

**Consultation delay:** the delay from the onset of first symptoms to first psychiatric consultation varied from 0 to 120 months (10 years). The average consultation delay was 15 months (±23.0) with a median of 6 months. The delay was more than 6 months in around half of the cases (48.3%). The symptoms that motivated the first consultation were hallucinations (43.5%), followed by sleep disorders (37.5%) and aggressive behavior (36.2%) (Table 2). The main reason of delayed consultation was lack of knowledge about psychiatric symptoms in 56.4% of cases followed by illness beliefs in 25.7% (Figure 2).

**Table 2:** Symptoms motivating the first consultation in psychiatry in patients with SMI

Symptoms	Number	Percentage
Hallucinations	101	43.5
Sleep disorder	87	37.5
Agressive behaviour	84	36.2
Incoherent speech	61	26.3
Bizarre behavior	52	22.4
Decline in functioning	38	16.4
Fugue	28	12.1
Character disorder	24	10.3
Substance abuse	2	0.9

**Factors associated with delayed psychiatric counseling:** univariate analysis between the studied factors and the delay in consultation showed that the lack of aggressiveness was significantly associated with a delayed consultation (more than six months) with p=0.04 (Table 3). The multivariate analysis showed a significant relationship between aggressive behavior and non-delayed consultation with p=0.04; Standard Deviation at 95%: (0.3-0.9); odds ratio: 0.4.

**Table 3:** Univariate analysis between studied factors and delayed consultation in patients with SMI at Department Psychiatry A, Razi Hospital

Factors		Consultation delay over 6 months		
		Number	percentage	P
Gender	Male	56	24.1%	0.2
	Female	56	24.1%	
Level of education	Illiterate	8	3.4%	0.2
	Primary	57	24.6%	
	Secondary	38	16.4%	
	Higher	9	3.9%	
Marital status	Single	68	29.3%	0.8
	Married	33	14.2%	
	Divorced	1	0.4%	
	Widower	10	4.3%	
Area	Urbain	94	40.5%	0.5
	Rural	18	7.8%	
Socio-economic level	Low	89	38.4%	
	Medium	22	9.5%	
	High	1	0.4%	
Symptoms	Hallucinations	45	19.4%	0.3
	Sleep disorder	48	20.7%	0.1
	Agressive behaviour	48	20.7%	0.02
	Incoherent speech	24	10.3%	0.1
	Bizarre behavior	26	11.2%	0.7
	Decline in functioning	19	8.2%	0.8
	Fugue	17	7.3%	0.1
	Character disorder	12	5.2%	0.8
	Substance abuse	2	0.9%	0.2
Diagnosis	Schizophrenia	65	28.0%	0.7
	Bipolar disorder	31	13.4%	
	Schizoaffective disorder	6	2.6%	
	Unspecified schizophrenia spectrum or bipolar disorder	10	4.3%	
Reasons	Ignorance of symptoms	59	42.1%	0.1
	Illness beliefs	14	10.0%	
	lack of aggressiveness	29	20.7%	
	stigmatization of psychiatry	5	3.6%	

## Discussion

This is the first study from Tunisia which describes the pathways of care in psychiatry and examines the consultation delay.

**Pathways to mental health care:** more than the third of the study population (34.1%) used traditional methods in the first place. This result is in accordance with pathway-to-care studies done in Africa [20-25,31,32] and Asia [18,30,33], where non-physicians (especially traditional and religious healers) formed a significant proportion of first carers. Different cultural effects can influence causal explanations of SMI between patients and different ways of searching treatments (acupuncture, consultation with a medium, herbal treatment, consultation with religious healers) [34]. According to a study conducted in Tunisia assessing beliefs and attitudes of relatives of patients with schizophrenia, religious causes were found in76.9% of cases. The participants first cited religious explanations (God's will or fate, God's punishment) then magical ones (witchcraft and possession by "djinns") and around the third of the participants believed in non-medical practices such as reading Holy Koran verses, charity and exorcism [35,36]. According to the results of the present study, one fifth of the cases (19.0%) had recourse to a GP. In Tunisia like in other African countries, the majority of GPs (which represents a large part of non-specialists) are not well enough trained to properly diagnose and manage mental illness [15]. In Tunisia, training of the physicians in primary health care centers is an ongoing process [17]. Thus, this process includes only those who applied for such training which makes it insufficient. The lack of staff combined with the huge need for care are principal limits for this training [15].

In Tunisia, specialist mental health care services are very unevenly distributed over the territory. So, most people seeking mental health care turn to Razi Hospital in the capital, which is the only specialized hospital in mental health care in the country. Outside of the capital, people turn to the few psychiatric units attached to general university hospitals or to very limited psychiatric units in the regional hospitals [15]. The present study confirmed these findings, where a little less than one fifth of the cases (17.7%) went to Razi Hospital, a very low part of them (2.1%) visited a psychiatrist in a general hospital and the one fifth (21.1%) visited a psychiatrist in private practice. The big resort of patients to the private sector of health care services is an important finding in the present study. In fact, one third of the cases consulted directly the private sector. This could be explained by the difficulties of access to public health care services as mentioned, but also by the cultural and social representation of psychiatric illness in Tunisia [37].

**Consultation delay:** the most frequent symptoms that motivated the first consultation in psychiatry in the present study were hallucinations followed by sleep disorders and aggressive behavior. This result is in congruence with literature. In fact, hallucinations are very common among patients with psychosis, and repeated exacerbation of symptoms like hallucinations and disturbed behavior often characterize the early stages of psychosis [38]. The majority of patients with psychosis or schizophrenia are diagnosed in their first episode of illness although the literature suggests that some of them may have had psychotic experiences for many years without consulting [11]. In fact, the "prodromal" period of SMI is typically characterized by some deterioration in social and professional functioning, as well as sub-threshold mood or psychotic symptoms. When the first full-blown psychotic or mood episode is resolved, symptoms may disappear or reduce. However, some symptoms often persist, which tend to interfere with the person's ability to return to study or to work [9]. In recent years, the early detection of and intervention in psychosis have proved their efficiency to delay or possibly prevent their onset [39]. Consequently, mental health promotion programs and policies should focus on people’s awareness about symptoms of SMI especially during the prodromal period or the first episode [40-42]. The present study showed that the average consultation delay was 15 months (±23.0) with a median of 6 months. This delay is considered as very long compared to the mean total delay of 16.3 (±21.3) weeks worldwide [42].

According to the literature, the delay between the onset of the first symptoms of psychosis and the first consultation in psychiatry varies across the world from less than a week (in Cuba, Central America, Romania, Spain, Serbia and Czech Republic) [29], to 3 months (in Turkey and Poland), 6 months (as in Bangladesh, Mexico and Mongolia) [30] and more than 6 months in other countries (in India) [33]. The main reason for delayed consultation among the studied patients was lack of knowledge about psychoses in more than half of the cases, followed by illness beliefs in one quarter. This is in consistence with the literature where knowledge about these disorders plays a crucial role in determining the delay between the onset of illness and demand for help [29]. The impact of culture and social support on recognition of mental health problems and the speed of pathways to care has also been confirmed [28]. This may explain the important part of consultations within the private sector in our study, which is socially better accepted and less stigmatizing than consulting at a mental hospital. A recent qualitative study of factors responsible for the delay in treatment in patients with SMI showed that the most common factor responsible for treatment delay was unawareness of illness and most of the interviewed family members had no knowledge about the patient's illness at the beginning [43]. These findings underline the need for public education and awareness about psychotic and bipolar disorders. Improving awareness is one important way of ensuring early detection [44]. Nevertheless, insidious onset and lack of aggressiveness was the third reason for consultation delay among 13% of cases. In fact, the negative symptoms are less well known and less disruptive to those close to people living with schizophrenia and to society in general [45]. The same is true for depressive episodes, which often initiate bipolar disorder and are less disruptive than manic episodes, but also less recognized as mental illness [42].

**Factors associated with consultation delay:** the results of this study showed that there was no significant relationship between the socio-demographic profile of studied patients (age, gender, level of education, marital status, area and socio-economic level) and the delay in consultation. The literature about this point shows considerable differences among countries. If the finding of the present study was consistent with those in Pakistan [46], in Nigeria [20], it was not the case in other studies where a significant relationship existed between education and socioeconomic levels and consultation delay, such as in South Africa [25], Ethiopia [21] and Ghana [22]. However, the lack of aggressiveness among first symptoms was significantly related with delayed consultation. In fact, when the first signs of mental illness are severe, including aggressive or violent behavior, people reach rapidly specialized services [24].

**Strengths and weaknesses of the study:** this study has a number of limitations. This was a cross sectional survey including patients consulting the Department of Psychiatry A of Razi Hospital, which can’t be a true representation of the national sample. There may be a recall bias while responding to the questionnaire. However, this study provides precious data in an understudied population. To the best of our knowledge, this is the first study in Tunisia exploring pathways to mental health care. The pathways to care were assessed by a standardized measure, The "Pathway to Mental Health Questionnaire" designed by the World Health Organization (WHO). The sample size is large compared to other studies.

## Conclusion

This study showed that a high proportion of patients consulted a traditional healer in the first place, whereas only about one fifth of patients consulted a general practitioner. More than 40% of patients accessed psychiatric services directly, a significant part of them accessing third line services in the first place. The consultation delay was very long and the main reason being a lack of knowledge about signs and symptoms of SMI. The principal recommendations we could draw from our study are to strengthen public education and awareness about SMI in the Tunisian population, to enhance the training in mental health of primary care providers, to facilitate access to specialized mental health care services and to implement an early detection program for SMI. The current study offered a first overview over the pathways to mental health care and the consultation delay in patients with SMI in Tunisia. Further research on this subject is needed in Tunisia to enrich the findings of this study.

### What is known about this topic

- Understanding the pathway to mental health care provides information about health services use among patients and helps to determine the care delay in psychiatry;
- The delay of consultation in psychiatry varies across the world from less than a week to more than 6 months and many factors may interfere with this delay;
- Reducing delays in accessing services and providing early intervention are key strategies in preventing morbidity and mortality associated with severe mental illness.

### What this study adds

- As in many sub-Saharan countries, a large proportion of the Tunisian population went to see traditional healers in the first place for their mental health problems;
- The average consultation delay for people with severe mental illness in Tunisia was 15 months with a median of 6 months which is considered very long;
- The main reason for delayed consultation among the studied patients was lack of awareness about signs and symptoms of severe mental illness, as well as social beliefs about origins of mental illness calling for a strengthening of public awareness and education.
